# Mechanism of Oleic Acid-Mediated Sulfur Vacancy Healing in Monolayer WS_2_

**DOI:** 10.1021/acsnanoscienceau.5c00091

**Published:** 2025-09-25

**Authors:** Leon Daniel, Dedi Sutarma, Osamah Kharsah, Charleen Lintz, Henrik Myja, Peter Kratzer, Marika Schleberger

**Affiliations:** † Faculty of Physics and CENIDE, 27170 University of Duisburg-Essen, Duisburg 47057, Germany; ‡ Faculty of Engineering and CENIDE, University of Duisburg-Essen, Duisburg 47057, Germany

**Keywords:** tungsten disulfide, oleic acid, photoluminescence yield, defect passivation, density functional theory

## Abstract

We uncover the mechanism behind the enhancement of photoluminescence yield in monolayer WS_2_ through oleic acid treatment, a promising scalable strategy for defect healing. By inducing sulfur vacancies through thermal treatment and monitoring the changes in photoluminescence yield and emission spectra, we demonstrate that in contrast to super acids, oleic acid heals the sulfur vacancy by providing substitutional oxygen, instead of hydrogen. Using density functional theory calculations, we provide insight into the underlying mechanism governing the oleic acid-mediated sulfur vacancy healing process. Our findings suggest that effective defect passivation by oxygen doping can be achieved through chemical treatment, opening a pathway for oxygen doping in transition metal dichalcogenides. However, we also highlight the limitations of chemical treatment, which may only lead to small increases in photoluminescence yield beyond a certain point.

## Graphical abstract

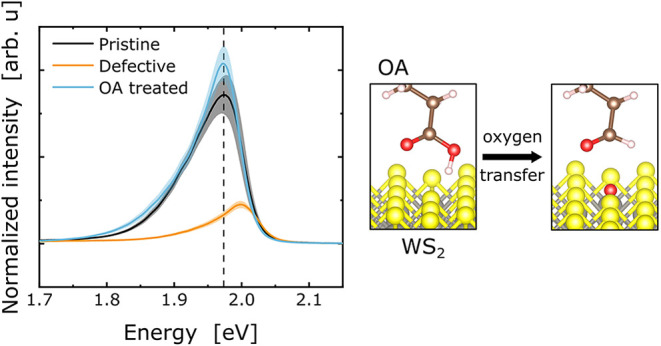

Transition metal dichalcogenides (TMDCs), particularly tungsten disulfide (WS_2_), are promising candidates for next-generation optoelectronic and valleytronic devices due to their unique two-dimensional (2D) nature and exceptional optoelectronic properties.^1,2^ As a direct bandgap semiconductor, monolayer WS_2_ has a bandgap of 2.4–2.7 eV,^3–5^ making it suitable for applications in the visible range.^5–7^ Its high photoluminescence (PL) yield^8–10^ enables a wide range of applications, from light emitting diodes (LEDs) to photodetectors.^11,12^ To develop WS_2_-based applications, precise control and manipulation of defects, particularly vacancies, are essential. Vacancies can significantly affect the electronic structure of TMDCs and modify their optical and electronic properties, allowing for customization of device functionalities.^13–19^

Point defects in WS_2_, such as sulfur vacancies, introduce flat defect states in the bandgap, altering the material’s native properties. These vacancies can act as a doping level or facilitate nonradiative recombination, affecting exciton recombination time and quantum efficiency (QE).^17,20–22^ To achieve specific material properties through defect engineering, both defect creation and passivation are crucial. Thermal treatment and ion irradiation have been used to induce vacancies in TMDCs, enabling precise control over defect type and density.^18,23–28^

Vacancy healing, on the other hand, remains a significant challenge. Chemical treatments, such as super acid TFSI,^29–34^ have been explored as an effective and scalable approach to restore vacancies and enhance performance. However, handling these chemicals can be difficult due to their corrosive nature.^35^ A benign-by-design chemistry is therefore desirable. Oleic acid, a relatively weak acid and harmless alternative, has garnered significant interest due to its ability to effectively passivate vacancies in WS_2_ and enhance photoluminescence properties by increasing the QE of TMDCs.^36–38^

For TFSI, it was first suggested that the acid’s sulfur heals sulfur vacancies in WS_2_.^33^ Then, deprotonation of the TFSI with three protons filling a sulfur vacancy was proposed.^31^ Recent experiments with Li-TFSI have shown that lithium can also fill the vacancy. This supports the idea that it is the cation that is responsible for the healing.^39^

In contrast to TSFI, a treatment with oleic acid preserves the n-doping characteristics as observed in field-effect transistors suggesting a different mechanism.^32^ It has been proposed that the −COOH ligand of oleic acid passivates the vacancy.^32,40–42^ However, no evidence has been provided for this hypothesis, and the exact mechanism of the defect healing with oleic acid thus remains unclear.

Our goal is to reveal the underlying mechanism of the vacancy healing process. We will also assess the efficacy of oleic acid in restoring the material’s pristine properties. To this end, we analyzed the evolution of vacancy density and band structure using in situ photoluminescence (PL). By combining experiments on pristine and defective WS_2_ with first-principles calculations, we found that the mechanism in oleic acid is indeed different from the mechanism in super acids. Oleic acid molecules act as an oxygen source, saturating sulfur vacancies in WS_2_ and removing defect in-gap states, leading to improved PL (as schematically depicted in Figure 1). Our work thus demonstrates the feasibility and limits of using chemical treatments for defect management in WS_2_, paving the way for optimized optoelectronic and valleytronic devices.

**Figure 1:**
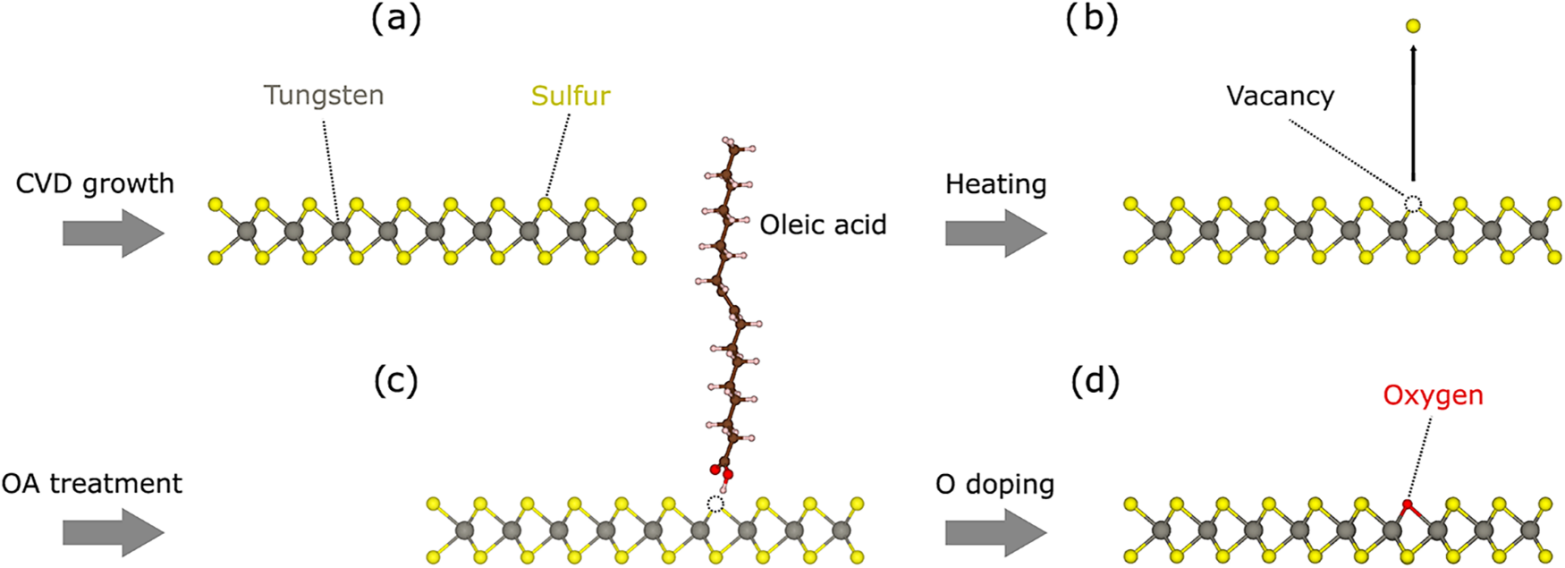
(a) WS_2_ grown by CVD is heated to introduce single vacancies (b). The defective material is then treated with oleic acid (c), which substitutes the vacancy with an oxygen atom (d).

Our study begins with a characterization of pristine WS_2_ samples grown by chemical vapor deposition (CVD, for details see Methods section). An optical microscopy image of a typical triangular monolayer flake is shown in (Figure 2a) together with the integrated PL intensity map revealing the typical grain boundaries. Starting from the center, the PL intensity increases outward, i.e., along the direction of growth of the flake. This is indicative of strong oxygen doping^43,44^ as we use no hydrogen to bind excess oxygen. The substitution of vacancies with oxygen enhances PL emission by removing in-gap defect states, enabling nonradiative recombination.^21,22^ This is in agreement with density functional theory (DFT) calculations showing that a vacancy saturated with an oxygen atom is highly favorable (see Supporting Information and refs 21,22,43–45 ).

**Figure 2:**
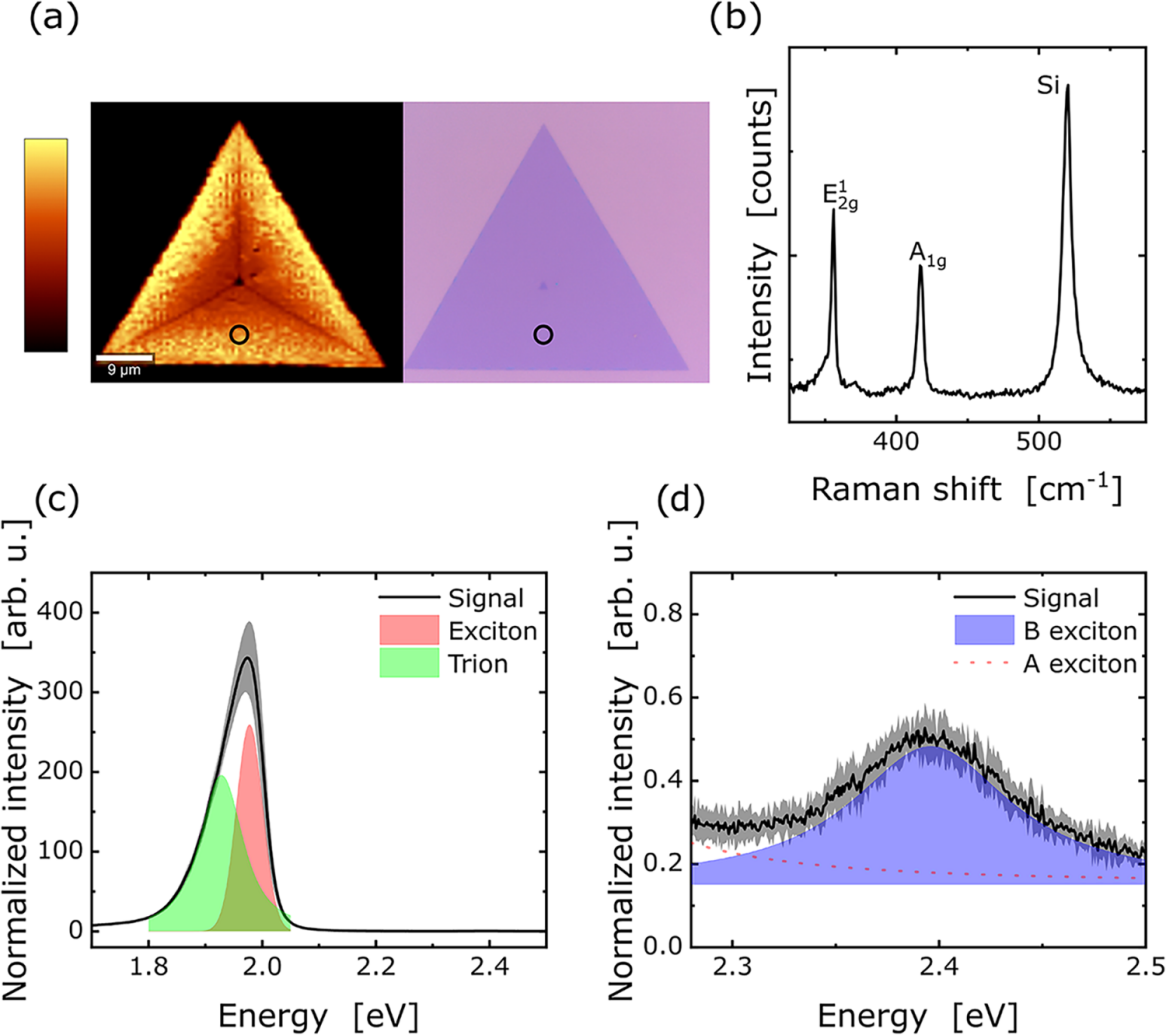
(a) Microscope image of a monolayer WS_2_ with corresponding PL map showing integrated intensity, with measurement locations marked. (b) Raman spectra with assigned Raman modes. (c) PL spectra of WS_2_ monolayer from 1.7 to 2.5 eV measured at 80 K, with fitted trion and exciton peaks. (d) Zoomed-in section of the PL spectrum, highlighting the smaller B exciton signal.

All further measurements were conducted at the spot with a strong PL intensity, marked with a black circle in (Figure 2a). Figure 2b displays a recorded Raman spectrum of the monolayer. By using a 457 nm laser, the 2LA (*M*) mode at 350.4 cm^–1^ is strongly suppressed, enabling a clear view on the E_2g_^1^ (Γ) mode at 356.8 cm^–1^ and A_1g_ (Γ) mode at 416.9 cm^–1^, confirming the monolayer.^46–48^

The pristine sample shows a strong emission at around 1.93 eV (Figure 2c), corresponding to the typical PL spectra for monolayer WS_2_.^2,6,7^ The PL signal is comparable to that of an exfoliated reference sample, indicating a low intrinsic defect concentration. The internal QE is estimated to be around 0.1% (see Methods section). The peak is asymmetric, suggesting the presence of trions.^49^

The zoomed-in section of the PL spectra in (Figure 2d) features the less intensive B exciton. This is consistent with the literature, where the A exciton emission is about 1800 times stronger than the B exciton emission.^6,7^ We modeled the band structure of a pristine WS_2_ monolayer via DFT calculations. Our calculations show a valence band splitting of 430 meV, which is 16 times higher than the conduction band splitting of 28 meV, consistent with literature values.^5–7^ The predicted energy difference of the optical transition (402 meV) matches our experimental value of 420 ± 7 meV measured at 80 K very well.

Next, we introduce sulfur vacancies by thermal processing, heating the sample to increasing temperatures and rapidly cooling it to 80 K for measuring between each step (Methods section). In (Figure 3a), the corresponding PL signal for the A-exciton is shown for selected temperature steps. The signal remains stable up to 450 K, consistent with other experiments.^27^ However, with further temperature increase, the signal intensity decreases significantly, dropping to 26.5% of the original intensity at 623 K as the data shows in (Figure 3b). Note that the intensity is normalized to a value of 100 for pristine WS_2_, which corresponds to a QE of 0.1%.

**Figure 3:**
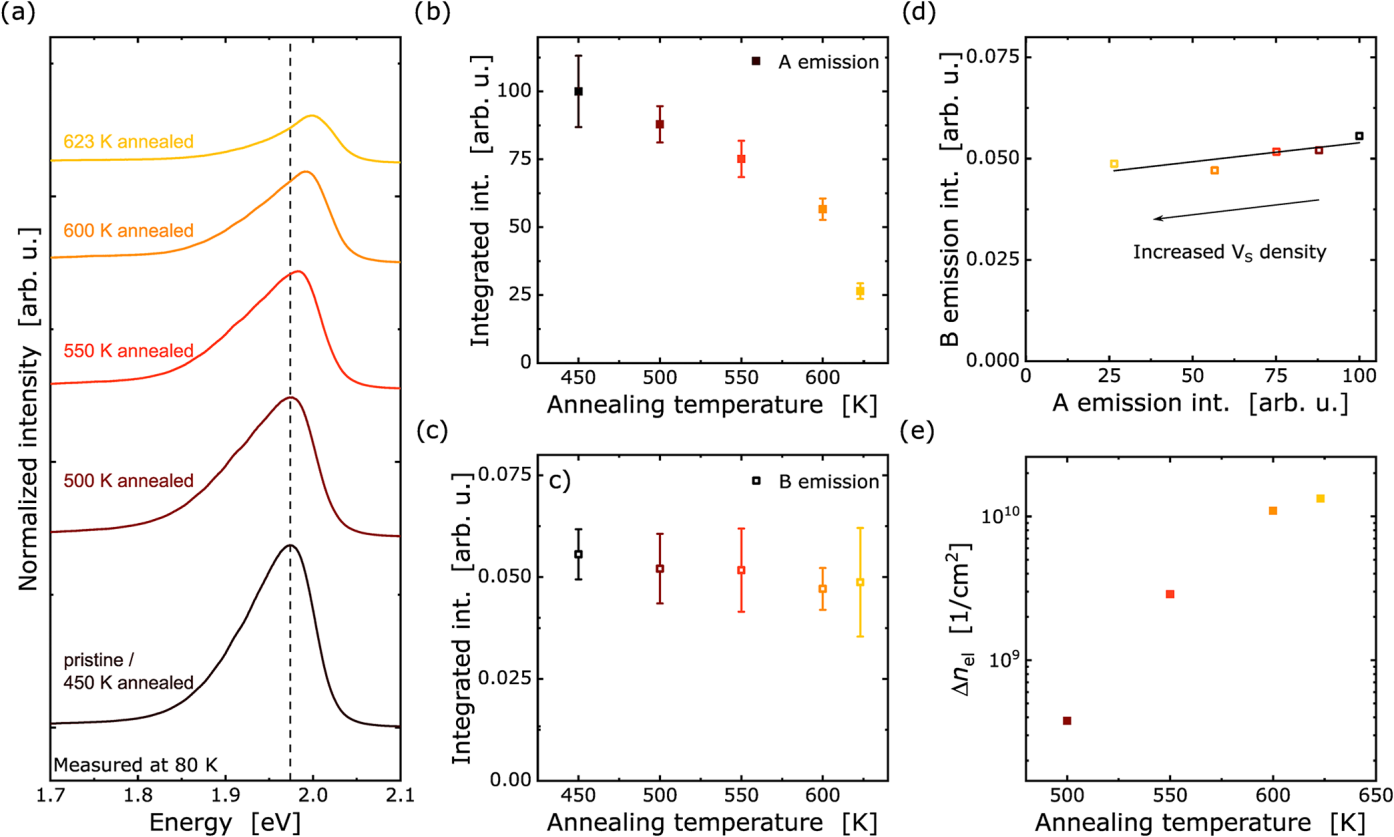
(a) Normalized PL spectra of WS_2_ for each heating step. (b, c) Signal intensity evolution of A and B emission with heating steps; PL intensity of pristine sample was used for normalization. (d) B emission vs A emission for the heated sample. (e) Evolution of free carrier density with increasing heating steps, calculated using the mass action model.

This is attributed to an increase in sulfur vacancies, which enable nonradiative recombination and reduce bright emission.^20,21^ Supporting DFT calculations show that the formation energy for a sulfur vacancy is 2.72 eV, significantly lower than other types of vacancies. Additionally, the reaction enthalpy for sulfur removal in the presence of oxygen is −0.09 eV, indicating enhanced sulfur vacancy creation.

The B exciton, emitted by a different optical recombination path, shows no significant change in emission, as seen in (Figure 3c). This suggests that the radiative recombination of the B exciton is unaffected by defects that influence nonradiative recombination of the A exciton. The ratio between A exciton emission and B exciton emission is often associated with defect density. For example, ion irradiation in MoS_2_ has been shown to decrease A exciton emission relative to B exciton emission.^13^ McCreary et al. proposed a linear dependency of the intensity ratio of B and A emission in TMDCs: *I*(*B*) = *b* + *a* × *I*(*A*) with *a* and *b* positive constants determined from WS_2_ samples with various defect densities.^50^ we have plotted the intensity ratio of A and B exciton emission in (Figure 3d). We determine *a* = 0.00015 ± 0.00005, and from the intersection with the *y*-axis *b* = 0.015 ± 0.002.

To check if the correlation between A and B excitons is universal or defect-type-dependent, we compared the correlation in a sample irradiated with different fluences of 100 eV argon ions, which increases in-gap states.^51^ The correlation differs significantly, and can be described by *I*(*B*) = 0.0006 + 0.024 × *I*(*A*), see SI. This suggests that individual defects can be differentiated based on the type of correlation between A and B excitons. Additionally, increasing sulfur vacancies should decrease the A exciton signal to almost zero while only slightly affecting the B exciton, indicating no large conversion from B excitons to A excitons.

With increasing vacancy density, the emission peak becomes increasingly asymmetric, attributed to increased trion emission at higher heating temperatures (see SI). This is consistent with the law-of-mass-action model, which describes the correlation between free charge carrier density and trion emission in TMDCs.^52–56^

1 $$\frac{N_{X}n_{\mathrm{el}}}{N_{X^{-}}}=\frac{4m_{X}m_{e}}{\pi \hbar^{2}m_{X^{-}}}k_{B}T\exp\left(-\frac{E_{b}}{k_{B}T}\right)$$

*N*_X_ and *N*_X^–^_ are the concentration of free excitons and trions, *m*_e_, *m*_X_ and *m*_X^–^_ are the effective mass of the electron, free exciton and trion, *k*_B_ and *T* are the Boltzmann constant and temperature, and *E*_b_ is the trion binding energy. We estimate the change in free charge carrier density Δ*n*_el_ with higher heating steps, as shown in (Figure 3e), which suggests a maximum increase of up to 1.3 × 10^10^ cm^–2^ for the final heating state. These carriers are attributed to ionized donor levels associated with S (or Se) vacancies.^57,58^

The signal position is analyzed to determine the splitting of conduction and valence bands. A significant shift in the A exciton maxima is observed with increasing defect density, as seen in (Figure 3a), while the B exciton remains largely unaffected. The A-B-exciton splitting is plotted for different heating steps in (Figure 4a). The origin of the band splitting is the spin–orbit coupling (SOC) induced mostly by tungsten.^59,60^ Sulfur vacancies have inconclusive effects on the band structure. However, some studies associate them with a red-shift due to higher trion emission^17^ and DFT calculations suggest a slight increase in the band gap of WS_2_.^21^

**Figure 4:**
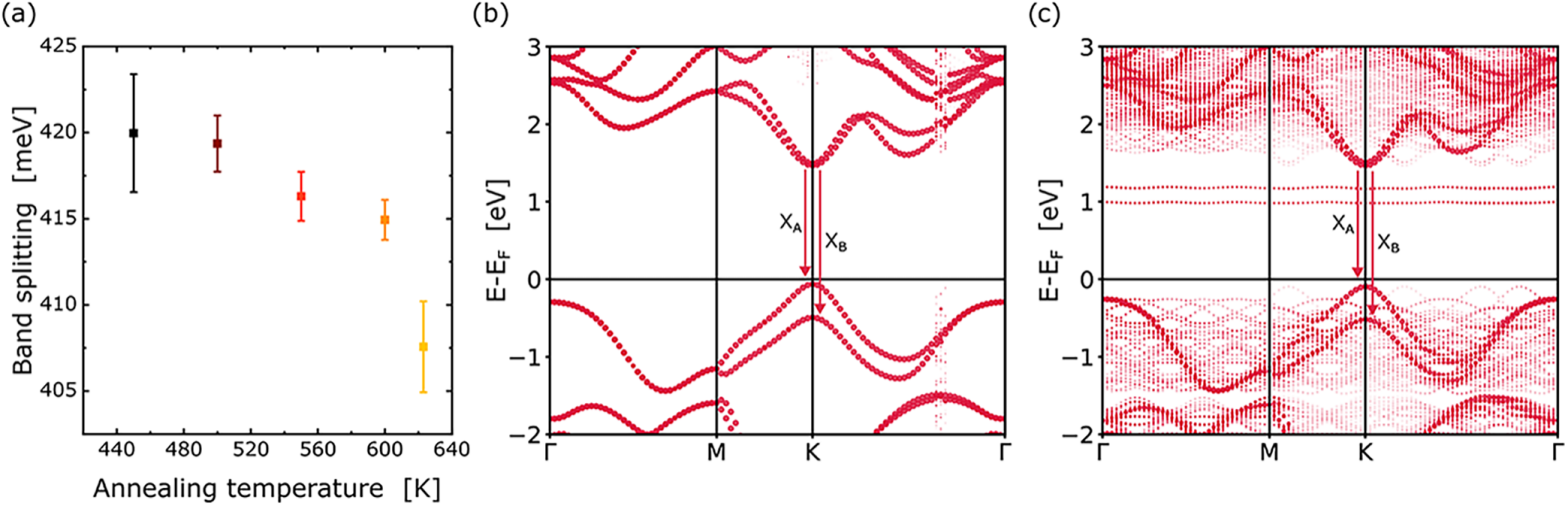
(a) Valence band splitting with increasing treatment temperature, with error bars representing one standard deviation. Band structure of WS_2_ in pristine condition (b) and with a V_S_ vacancy (c). The energetic difference between A and B emission (*X*_A_ and *X*_B_) decreases by 20.1 meV.

We analyzed the electronic band structure of WS_2_ with various defects (V_S_, V_WS_3__, O_S_). The results for pristine WS_2_ and V_S_ are shown in (Figure 4b,c). The vacancy induces two unoccupied states in the band gap and an occupied state in the valence band, leading to nonradiative recombination.^61^ The calculations also suggest a decrease in the energetic difference between A and B emission of 20.1 meV, comparable to the experimental value. With little change in B emission, this suggests a blue shift in A emission only. The removal of a WS_3_ cluster or oxygen substitution have only negligible effects, see SI.

In the following, we examine the effect of oleic acid treatment on restoring the PL signal in defective samples. After thermal treatment, the PL intensity drops and shifts to the blue (Figure 5a). However, oleic acid treatment significantly increases the PL intensity, consistent with previous reports.^32,36–38^ The A exciton emission increases by a factor of 4 on average, while the B exciton emission increases by 19%. This is attributed to the elimination of defect-induced in-gap states. The recovered PL emission of the A exciton exceeds the pristine sample’s by 8%. This might be due to a step in the oleic acid treatment, which involves washing in toluene. This might reduce contaminations present on WS_2_, enhancing electron–hole recombination. We also observe a slight reduction in doping concentration by up to 1.12 × 10^10^ cm^–2^ and a shift in A emission spectra back to its original position (B emission remains unaffected).

**Figure 5:**
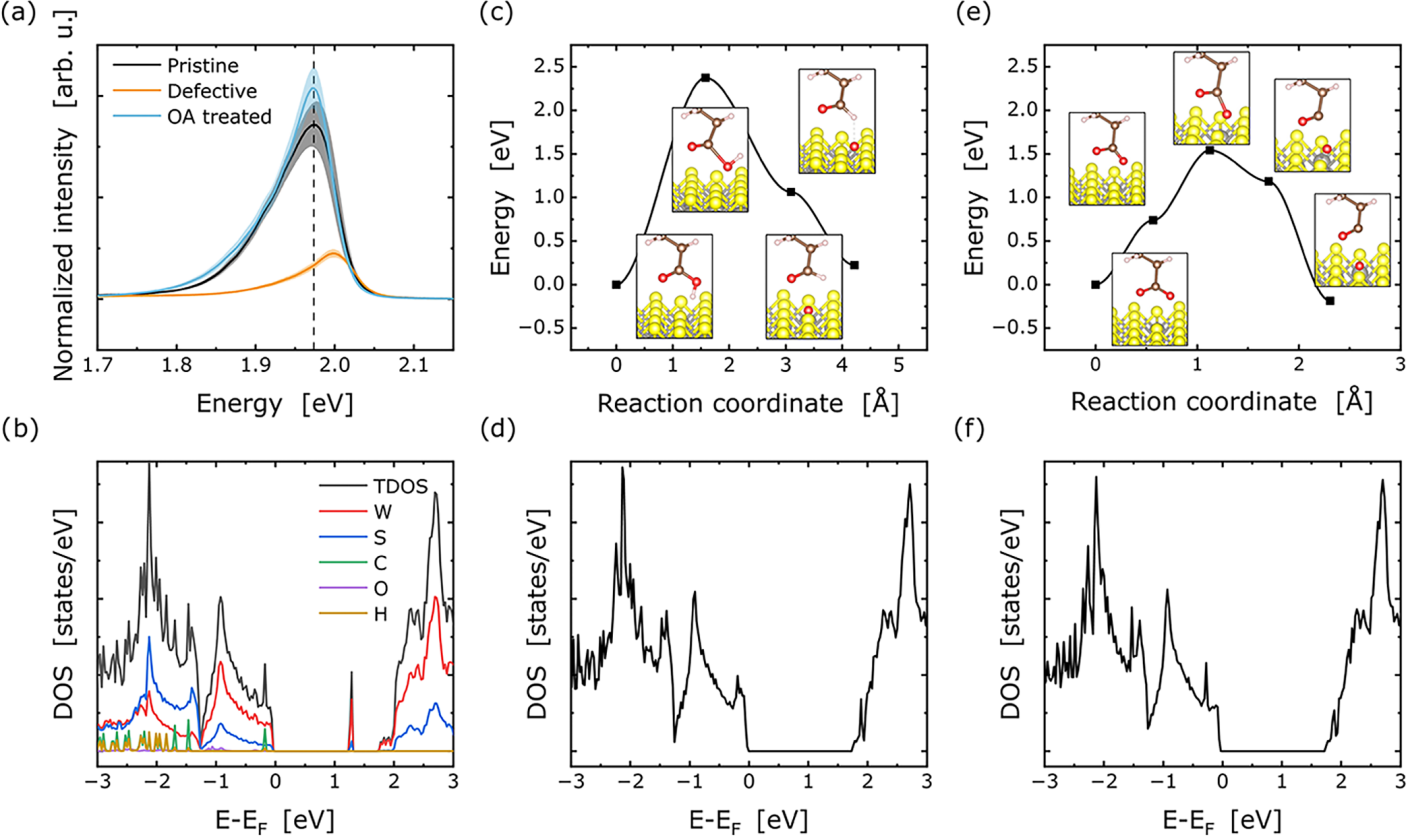
(a) Normalized PL signal at 80 K of a pristine CVD-grown sample after heating to 623 K and subsequent oleic acid treatment. (b) Element-resolved density of states (DOS) of oleic acid adsorbed on a sulfur vacancy in WS_2_. (c) Reaction path of oleic acid with corresponding DOS (d) at the final reaction step. (e) Reaction path of deprotonated oleic acid with corresponding DOS (f) at the final reaction step.

To investigate any possible doping effects, we analyzed the Raman spectra carefully, but did not observe any changes (see SI), which is in agreement with previous reports.^32^

We found that oleic acid treatment of pristine WS_2_ samples did not significantly improve PL emission, unlike previous reports. We attribute this to oxygen atoms occupying sulfur vacancies, removing in-gap states and preventing oleic acid from binding to or reacting with them. This suggests that oleic acid’s presence alone does not enhance PL emission, contradicting a purely physical interaction mechanism as, e.g., charge transfer resulting in reduced trion formation. We suggest that PL recovery is due to oxygen atoms provided indirectly during oleic acid treatment, which saturate V_S_ sites. This mechanism is supported by the fact that only point defects, not larger defects (see SI), can be healed. Note that this is a critical limitation of the method in general.

While O_2_ molecules might be present on the surface, they do not dissociate readily at V_S_.^44,62^ Therefore, another source for oxygen must be involved. To support our claim, that the oleic acid provides the oxygen, we performed DFT calculations on the reduction of oleic acid to a fatty aldehyde, releasing an oxygen atom. (Figure 5b,5c) display the calculated minimum energy path for the reduction of oleic acid and deprotonated oleic acid at V_S_, respectively. While the former reaction is found to be endothermic by 0.22 eV, the latter is exothermic by 0.19 eV. As shown in (Figure 5b), oleic acid reduction proceeds by elongation of the bond between the hydroxy group and the acyl head, with an energy barrier of 2.37 eV. The reduction of deprotonated oleic acid is more likely, see (Figure 5c): After breaking the carbon–oxygen bond, the oxygen atom inserts itself into the sulfur vacancy, with an energy barrier of 1.54 eV. We note that thermal activation suffices to overcome the barrier of 1.54 eV even at room temperature. Eventually, we verified that for both reactions, the in-gap states associated with V_S_ are indeed removed. This is concluded from (Figure 5e,f), showing the calculated density of states in the final state of the reactions.

Previous studies with superacids such as TFMS^63^ or TFSI^30,31,34^ suggest hydrogen transfer as a mechanism for enhancing PL yield (for a review see e.g., ref 29). However, oleic acid is known to be a weak acid (p*K*_a_ 9.85 ^64^), making hydrogen transfer a priori less likely in this case. Also, the characteristic p-doping due to protonation is not observed with oleic acid,^32^ while on the other hand, oxygen is know to affect the doping only slightly.^21^ Thus, different mechanisms are likely to be at work in superacids as compared to oleic acid.

Note, deprotonation at V_S_ (see SI) could still play a role as a first step followed by reduction to the fatty aldehyde, as described above. Further, our DFT calculations (see SI) show that oleic acid, both in its integral or deprotonated form, can adsorb either on-top of or inside the sulfur vacancy, but in all four geometries studied by us, the passivation of the dangling bonds was incomplete. Thus, ligand passivation as suggested by Tanoh et al.^32^ can also be ruled out. We note that other chemicals, in particular sulfur-containing ones, e.g., thiols,^65^ may provide an alternative route to PL recovery, as has been discussed in the literature.

In summary, we systematically investigated the impact of thermally induced vacancies on A and B emission through in situ measurements. We observed a strong effect on A excitons, while B excitons were almost unaffected by vacancy-induced defect states, suggesting that the correlation between the two excitonic signals is defect-type dependent. The energetic difference between A and B emission is influenced by the defect density. Our results demonstrate oleic acid’s effectiveness in restoring the material’s optoelectronic properties. We showed that oleic acid primarily saturates existing sulfur vacancies. Our calculations indicate that the PL enhancement observed with oleic acid is due to the introduction of substitutional oxygen, which heals vacancies and removes in-gap states. No further significant improvement beyond oxygen substitution could be achieved.

## Methods

### Sample Preparation

WS_2_ samples were grown via chemical vapor deposition (CVD) on SiO_2_/Si substrates. To prepare the growth substrates, an aqueous solution was mixed from 3.2 mL of ammonium metatungstate (AMT, Sigma-Aldrich), 2 mL of OptiPrep (Sigma-Aldrich), and 0.8 mL of DI water. This solution was spin-coated onto p-doped Si substrates with a 285 nm SiO_2_ layer,^66^ followed by spin-coating cholic acid sodium salt (Sigma-Aldrich) and subsequent annealing at 500 °C for 45 min to convert AMT to WO_3_.

The CVD growth was performed in a three-zone tube furnace with a quartz tube (ThermConcept) connected to an Ar source and exhaust, providing a flow rate of 500 sccm. The first zone was heated to 170 °C with 350 mg of sulfur (S powder, Sigma-Aldrich, 99.98%), while the growth substrates were positioned in the second zone at 725 °C for 60 min to form WS_2_. The third zone was kept at 600 °C to remove reaction byproducts.

Using transfer-free samples eliminates defects formed during transfer processes, such as localized strain caused by impurities on intermediate surfaces.^67^

### Thermal Treatment

The sample was placed in a Linkam THMS350V vacuum chamber, achieving a medium vacuum of 5 × 10^–3^ mbar. It was heated to various temperatures (up to 623 K) for 30 min, then rapidly cooled to 80 K using liquid nitrogen. PL measurements were taken at 80 K once equilibrium was reached.

### PL Measurements

PL measurements were performed using a Witec Alpha300 R setup. Spectra were averaged from 10 measurements with a 457 nm laser, focused to ≈ 830 nm spatial resolution. The full spectrum (1.5–2.7 eV) was taken to normalize the signal after background subtraction, using the Si substrate’s secondary Raman mode (985 cm^–1^). A constant laser power of 1.25 × 10^5^ W/cm^2^ was used, with the laser spot readjusted and refocused for each measurement to minimize signal fluctuations. All measurements were conducted at 80 K under vacuum conditions.

### Oleic Acid Post-Treatment

We followed Tanoh et al.’s instructions^32^ for oleic acid treatment. After heating, the sample was transferred to a nitrogen-filled glovebox and placed on a heating plate kept at 25 °C. Degassed oleic acid was carefully dropped onto the sample, covering the substrate. After 16 h, the oleic acid was washed away with toluene and dried with nitrogen.

### QE Measurements

For absolute QE determination of the CdSe/CdZnS core–shell nanoparticle reference, a home-built calibrated PL setup was utilized. A 15 cm diameter integrating sphere by Gooch and Housego is fiber coupled (Ocean Optics P200–2-UV–vis) to a HORIBA iHR320 monochromator equipped with a HORIBA Syncerity CCD. The detection system including the integrating sphere, fiber, monochromator and CCD are calibrated using an Ocean Insight HL-3 plus VIS-NIR calibrated light source. The sample is located in the center of the integrating sphere and is directly excited by a laser beam entering the sphere through one of the access ports. For excitation a tunable line with 10 nm bandwidth from a super continuum white light fiber laser (SuperK FIANIUM by NKT Photonics) was used. Corrections for differences in the excitation conditions have been applied by beam expansion and varying laser power by ND filters. With a reference measurement of a blank substrate reference and a measurement of the sample of interest, the QE is calculated from referencing the integrated calibrated intensity.^68^

### DFT Calculations

First-principles calculations were performed using Density Functional Theory (DFT) with VASP 6.3.0 version package.^69,70^ The interactions between ions and valence electrons were described using the projector-augmented wave (PAW)^71^ method, and electronic exchange and correlation were treated by the generalized gradient approximation of Perdew, Burke, and Ernzerhof (PBE).^72^ We employed a plane wave cutoff energy of 500 eV with an additional correction to the van der Waals dispersion interaction in the form of Becke–Johnson damping function D3.^73^ The material models including the defected system were built in a 6 × 6 × 1 supercell with 15 Å vacuum to avoid interaction with the periodic images, which corresponds to a defect density of 2.1 × 10^13^ cm^–2^. A 9 × 9 × 1 Monkhorst–Pack k-point grid was used to sample the 2D Brillouin zone. The unfolding of the band structure was computed using the VASPKIT code.^74^ We constructed the minimum energy path of our reactions using the Nudged Elastic Band (NEB) method.^75^

### Large Language Models

We used an LLM (Meta Llama 3.1 8B Instruct) to improve the linguistic quality of the manuscript. Neither this nor any other LLM was used to generate scientific content.

## Supplementary Material



## Notes

Raman spectra of pristine, annealed and OA treated WS_2_; PL spectra of CVD-grown and exfoliated WS_2_; B over A emission intensity for annealed and irradiated WS_2_; PL spectra for OA treated irradiated WS_2_ sample; PL spectra for OA treated pristine WS_2_ sample; PL spectra for annealed and air exposed WS_2_ sample; trion to exciton ration for heated WS_2_ sample; additional details for law-of mass action model and defect formation energy calculations; effective band structure of WS_2_ with O_S_, V_W_ and V_WS3_; details regarding simulated DOS of OA molecule on vacancy site; DOS for WS_2_ with V_WS3_ and OA molecule; DOS for protonated V_S_ (PDF)
