# Supplementary material for: Mechanism of Oleic Acid-Mediated Sulfur Vacancy Healing in Monolayer WS2
Source: ACS Nanosci Au. 2025 Sep 25;5(6):576–84. doi: 10.1021/acsnanoscienceau.5c00091 (PMC12715634; doi:10.1021/acsnanoscienceau.5c00091)
Supplement: Supplementary file 1 [file ng5c00091_si_001.pdf]

Supplementary Information

Mechanism of Oleic Acid-Mediated Sulfur Vacancy Healing in Monolayer WS_2

Leon Daniel[a], Dedi Sutarma[a], Osamah Kharsah[a], Charleen Lintz[a], Henrik Myja[b], Peter Kratzer[a], and Marika Schleberger[a]*

[a]Faculty of Physics and CENIDE, University of Duisburg-Essen, Duisburg, 47057, Germany

[b]Faculty of Engineering and CENIDE, University of Duisburg-Essen, Duisburg, 47057, Germany

E-mail: marika.schleberger@uni-due.de

Phone: +49 203 379 1600/1601. Fax: +49 203 379 2334

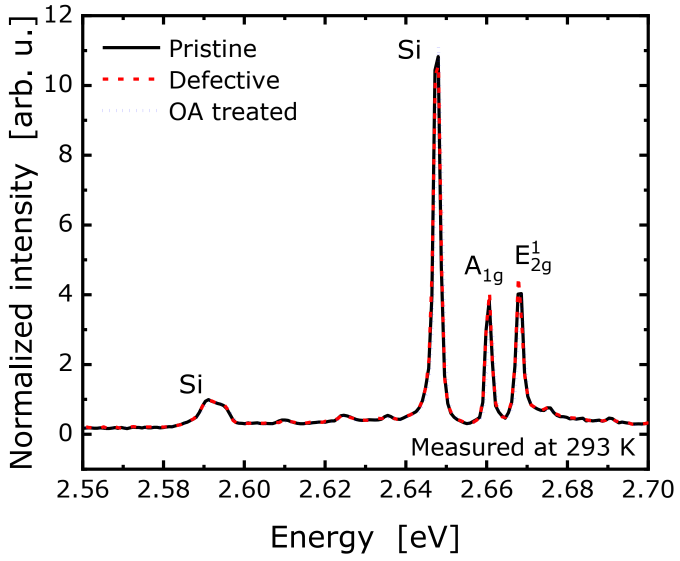

Figure S1. Normalized Raman spectra for WS_2 sample in pristine condition, after being annealed and after OA treatment.

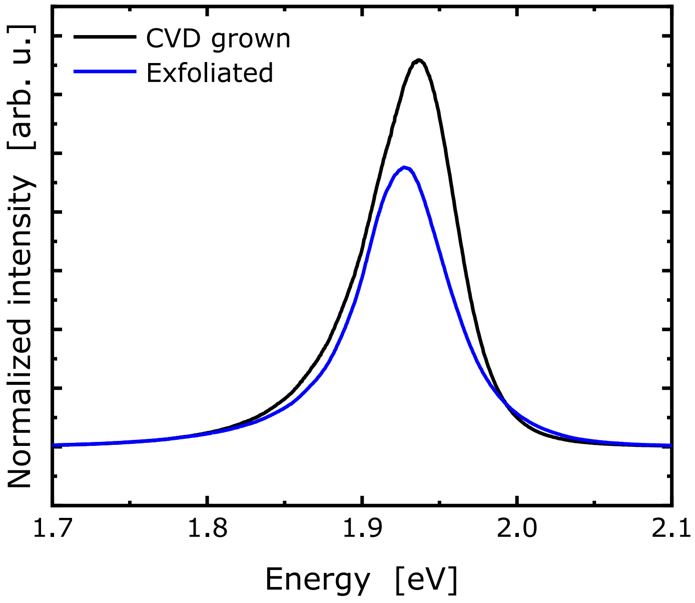

Figure S2. Normalized single PL spectra for WS_2 sample in pristine condition and for an exfoliated reference sample.

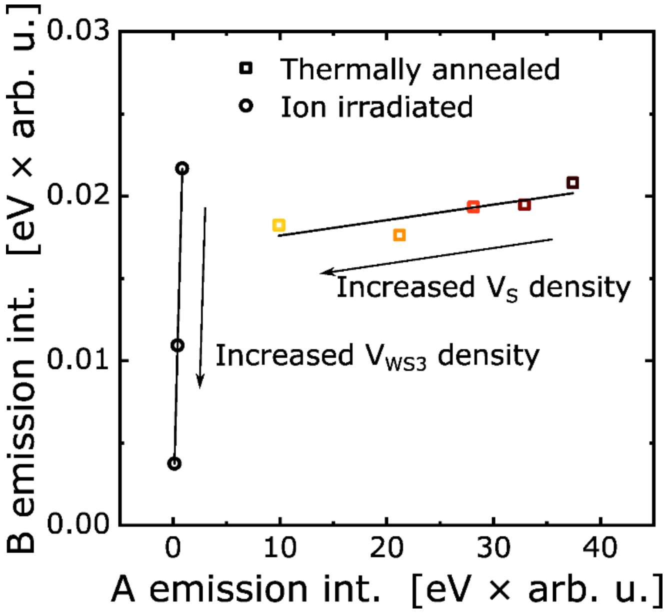

Figure S3. B emission intensity plotted over A emission intensity for annealed sample and ion bombarded sample.

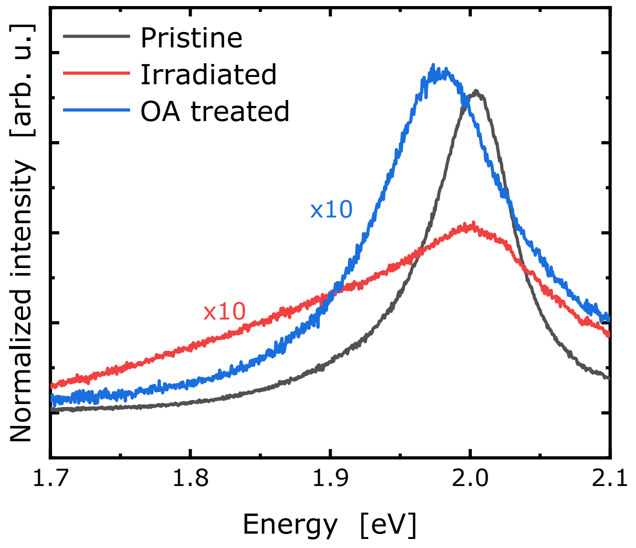

Figure S4. Normalized PL spectra for a WS_2 sample in pristine condition, after being bombarded with 100 eV argon ions and after being treated with OA measured at 80 K.

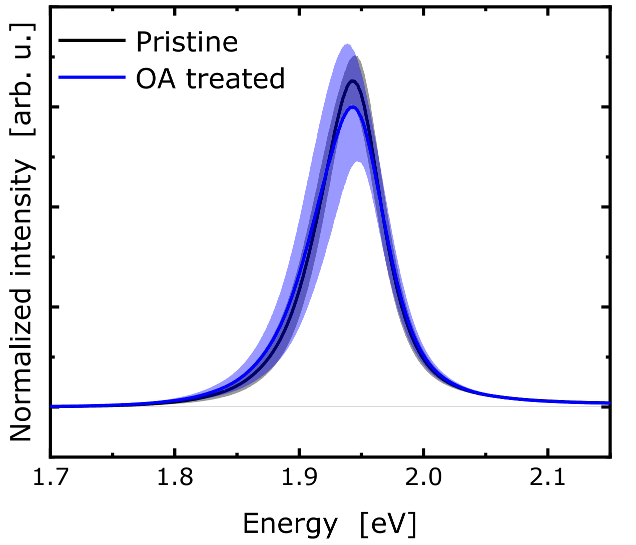

Figure S5. Normalized PL spectra for a WS_2 sample in pristine condition and after being treated with OA measured at room temperature.

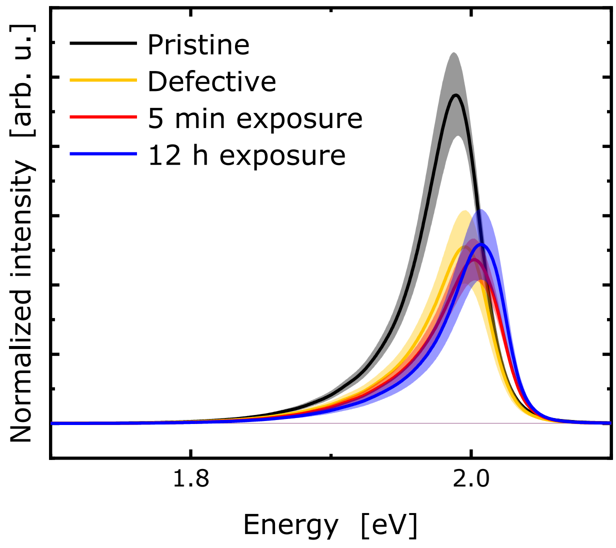

Figure S6. Normalized PL spectra for a WS_2 reference sample in pristine condition, after being annealed to 623 K and being exposed to air for 5 min and 12 h measured at 80 K.

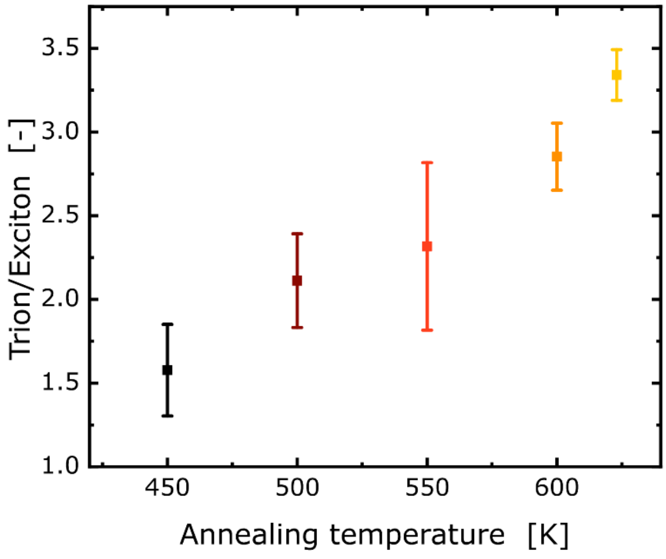

Figure S7. Trion to free exciton ratio for the annealed a WS_2 sample.

Law-of-Mass action model

We calculated the effective mass of the electron $m_e = 0.366m_0$, for the hole $m_X = 0.722m_0$ and for negative trion $m_{X^-} = 1.088m_0$, where m_0 is the mass of the free electron. For the trion binding energy we assume $E_b = 45$ meV from our measurements. With k_B being the Boltzmann constant, T the temperature ($80\ K$), $\hbar$ the reduced Planck constant, N_X and N_{X^-} the concentration of excitons and trions, we can derive from Equation 1, if a similar photon emission probability is assumed for the decay of the trion and free exciton, the following relation:

$$\frac{I_{X^T}}{I_{tot}} \approx \frac{2.44 \times 10^{-14} n_{el}}{1 + 2.44 \times 10^{-14} n_{el}}$$

where n_{el} is given in m^{-2}.

Defect Formation Energy

$$E_F = E_D - E_P - \sum_i n_i \mu_i$$

The defect formation energy E_F is obtained from the DFT calculations as the difference between total energy of defected system E_D and reference system E_P minus the chemical potential μ_i of the species. For the resulting value of E_F, the value of μ_i at zero temperature and pressure for sulfur, tungsten, and oxygen were calculated from the bulk structure of each species.

On the other hand, the enthalpy of SO_2 formation from the sulfur released from the WS_2 is calculated using DFT according to the following reaction

$$WS_2 + xO_2 \rightarrow WS_{2-x} + xSO_2$$

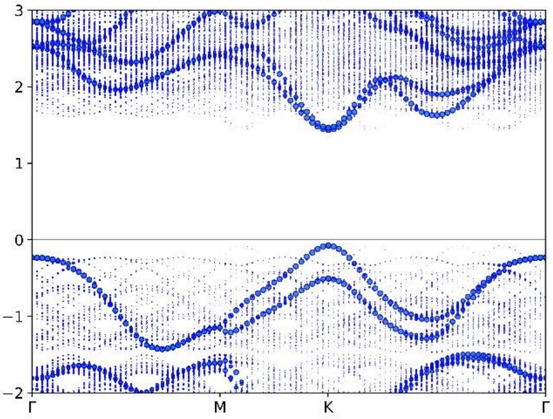

a)

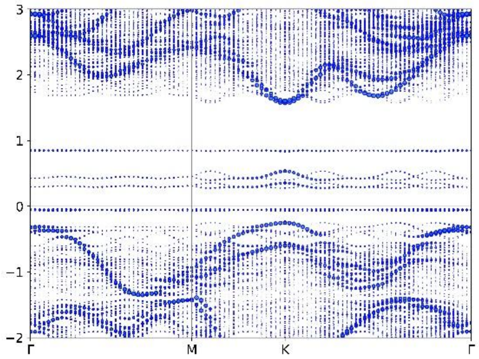

b)

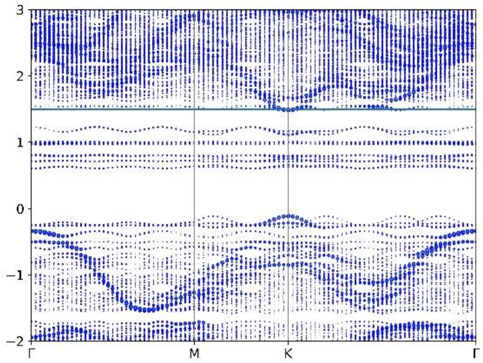

c)

Figure S8. Effective Band Structure (EBS) of WS_2 with a) O_s, b) V_W, and c) V_{WS3}

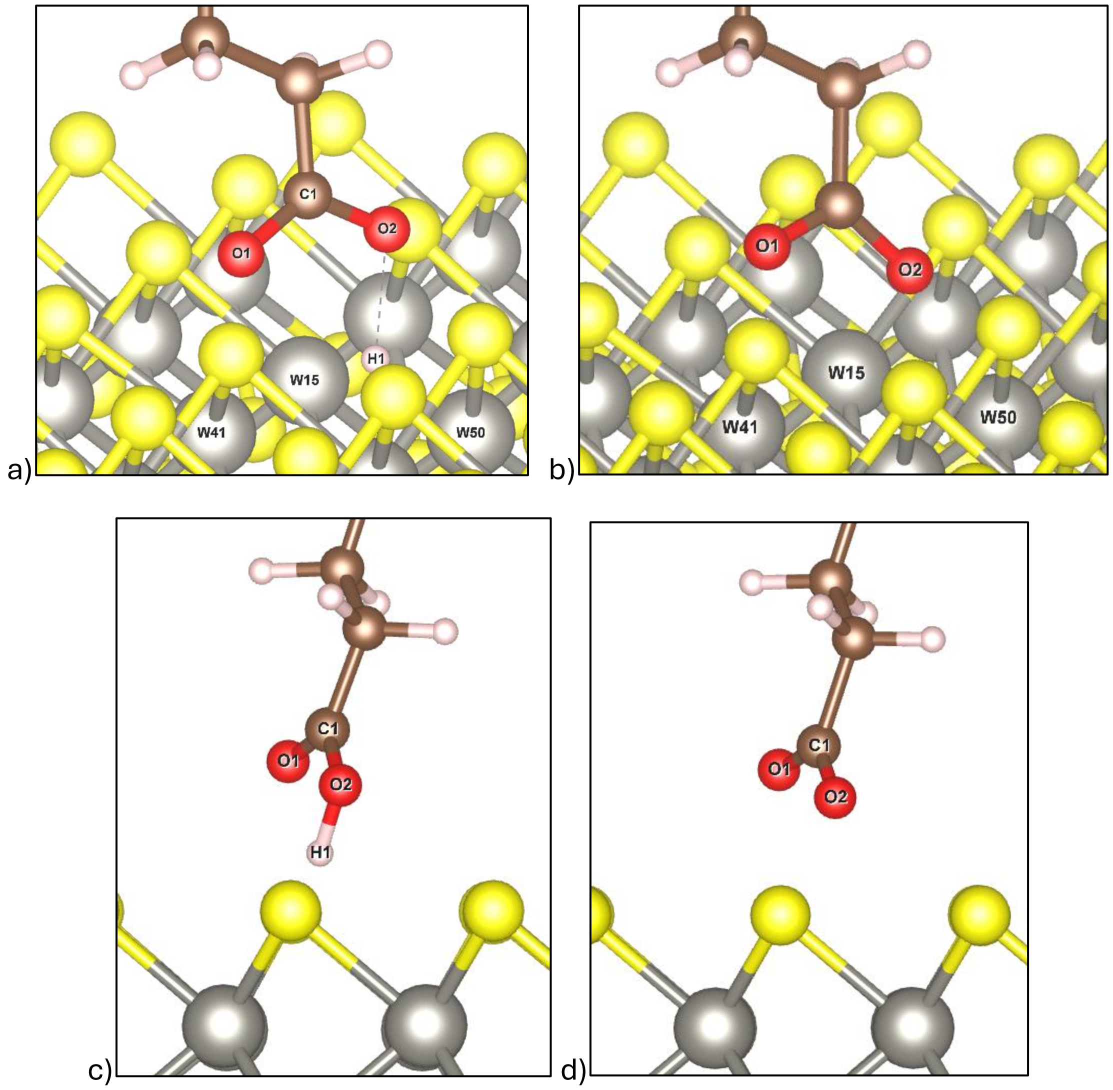

Figure S9. 4 models of oleic acid interacting with WS_2 embedded in the V_s site; a) OL1 and b) OL2, and at 1 Å distance from the WS_2; c) OL3 and d) OL4. Both OL2 and OL4 have the deprotonated form of oleic acid.

Table S1. Structural comparison of oleic acid with WS_2 models

Model	O1-C1-O2 (°)	O2-H1 (Å)	S-W41-S (°)
Oleic acid	119.97	0.96	
WS_2 with V_s			81.40
OL1	124.49	1.64	85.03
OL2	116.05		80.36
OL3	122.67		87.64
OL4	128.45	0.99	81.63

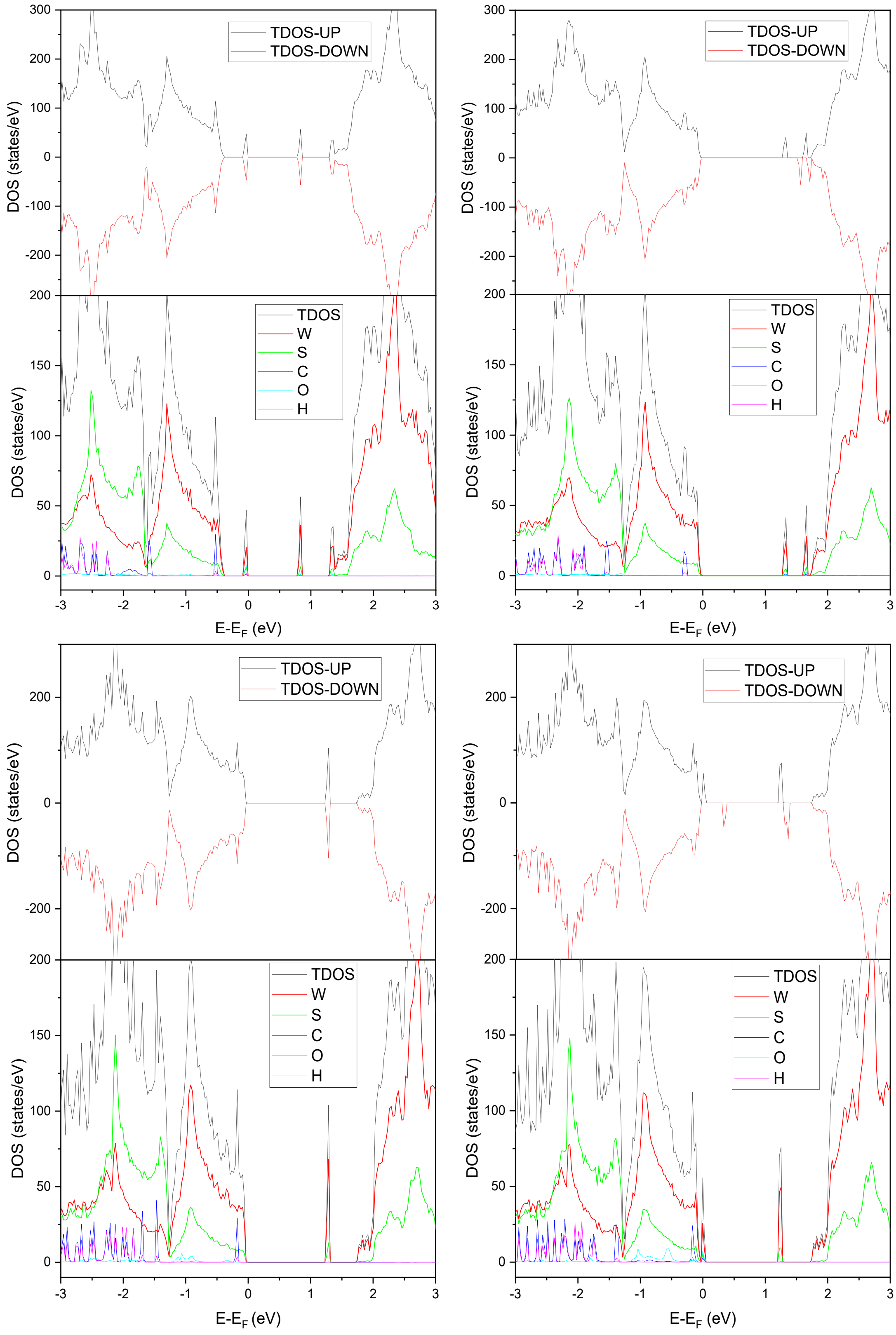

Figure S10. Density of States (DOS) of OL1, OL2, OL3, and OL4, showing the persistent presence of in-gap states in all four models.

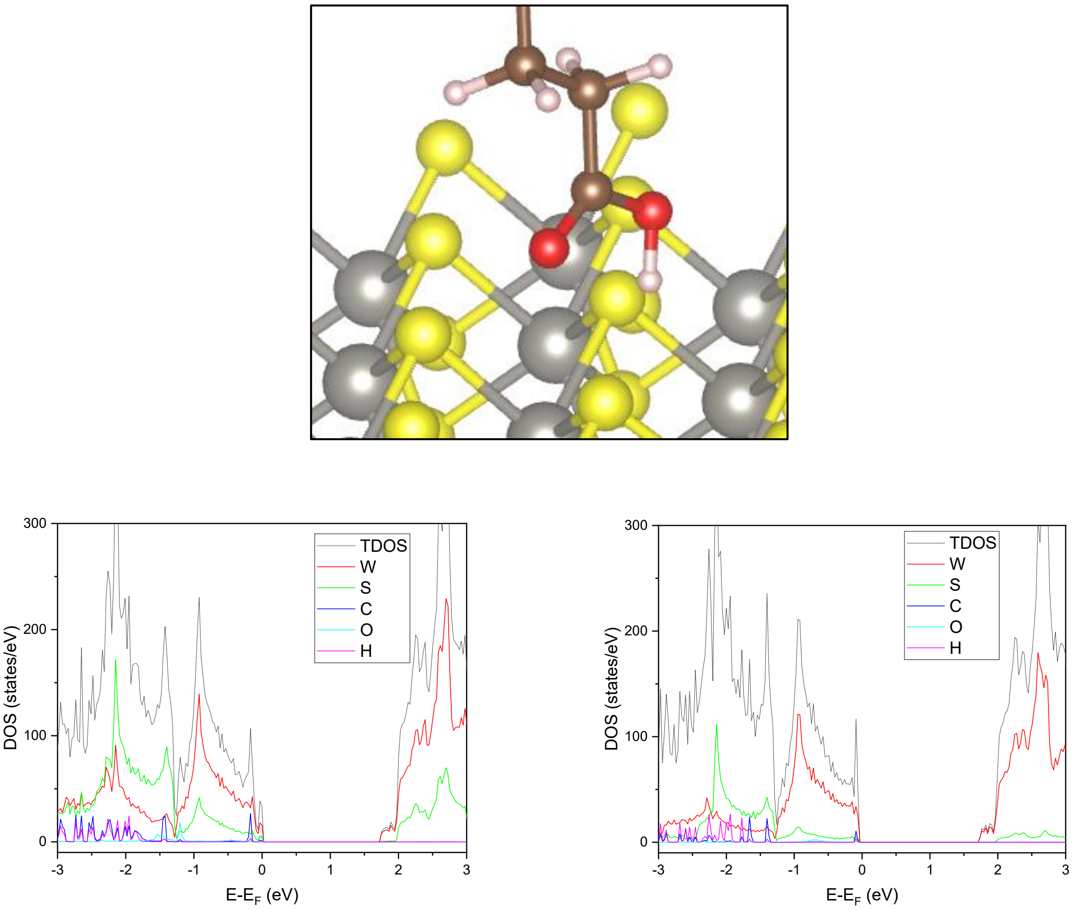

Figure S11. Control test of OL3 and OL4 models with WS_2 pristine. The oleic acid in both forms sitting on top of WS_2 surface yields no in-gap state.

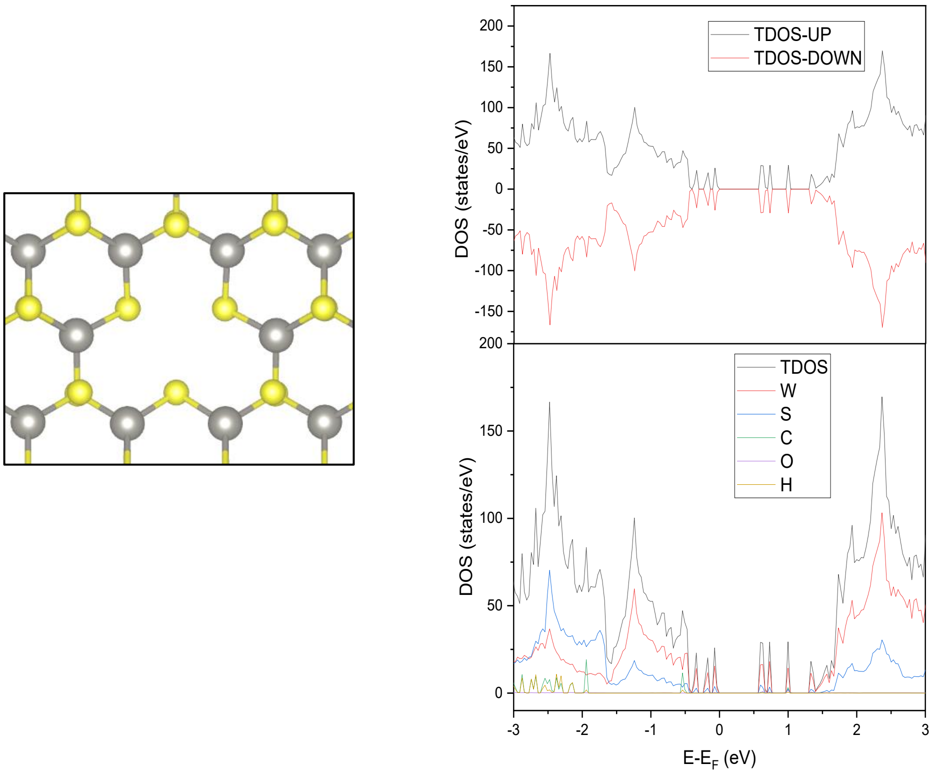

Figure S12. Density of States (DOS) of cluster vacancy (V_{WS3}) with the presence of oleic acid

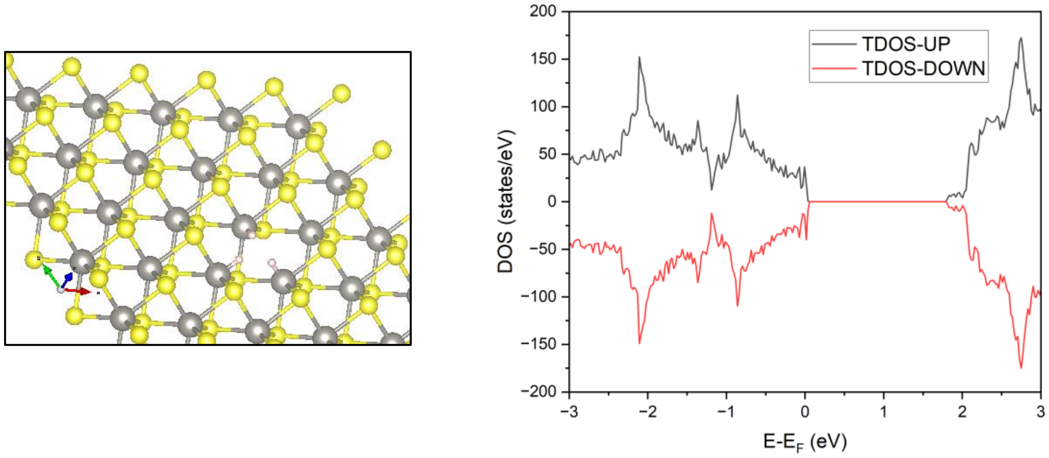

Figure S13. Saturation of V_s on WS_2 with 3 hydrogen atoms. The figure on the left show the relaxed structure of this model with each hydrogen atoms saturating tungsten dangling bonds. DOS calculation shows the elimination of signature V_s in-gap state.
